# Can neutrophil to lymphocyte ratio predict lamina propria invasion in patients with non muscle invasive bladder cancer?

**DOI:** 10.1590/S1677-5538.IBJU.2016.0158

**Published:** 2017

**Authors:** Haci Ibrahim Cimen, Fikret Halis, Hasan Salih Saglam, Ahmet Gokce

**Affiliations:** 1 Department of Urology, Sakarya Training and Research Hospital, Sakarya University, Sakarya, Turkey

**Keywords:** Urinary Bladder, Neoplasms, Inflammation

## Abstract

**Objective:**

Recent studies have demonstrated the role of systemic inflammation in the development and progression of cancer. In this study, we evaluated whether preoperatively measured neutrophil-to-lymphocyte ratio (NLR) can predict lamina propria invasion in patients with non-muscle-invasive bladder cancer (NMIBC).

**Material and Methods:**

We reviewed the medical records of 304 consecutive and newly diagnosed patients with bladder cancer who had been treated with transurethral resection between January 2008 and June 2014. In total, 271 patients were included in the study and the patients were divided into two groups according to the pathological stage (Group 1: Ta, Group 2: T1). NLR was calculated by dividing the absolute neutrophil count (N) by the absolute lymphocyte count (L).

**Results:**

In total, 271 patients (27 women and 244 men) were enrolled. Mean age was higher in Group 2 than in Group 1 (67.3±10.8 vs. 62.9±10.8, p<0.001). Furthermore, the presence of high grade tumors and tumors ≥3cm in size was statistically higher in Group 2 than in Group 1 (70.9% vs. 9.9%, p=0.0001; 71.8% vs. 36%, p=0.0001, respectively). While the mean white blood cell (WBC) and N counts were statistically insignificant (7.63±1.87 vs. 7.69±1.93, p=0.780; 4.72±1.54 vs. 4.46±1.38, p=0.140; respectively), L was significantly lower and NLR was significantly higher in Group 2 than in Group 1 (2.07±0.75 vs. 2.4±0.87, p=0.001; 2.62±1.5 vs. 2.19±1.62, p=0.029; respectively).

**Conclusion:**

Our data indicate that high NLR and low L are statistically associated with T1 stage, whereas low L are able to predict lamina propria invasion in patients with NMIBC. These findings suggest that pretreatment measurement of NLR may provide valuable information for the clinical management of patients with NMIBC. Prospective studies are now required to further validate the role of NLR as a risk factor in NMIBC*.*

## INTRODUCTION

Bladder cancer is the most common urinary tract malignancy and the third most common cancer in men, following prostate and lung cancer, with an estimated 58.950 new cases and 11.820 deaths in 2016 alone (1). While 75%-85% of the patients with bladder cancer present with a disease confined to the mucosa (stage Ta, CIS) or submucosa (stage T1), the remaining cases may often include bladder cancers that invade the muscle (2). Moreover, it is known that non-muscle-invasive bladder cancer (NMIBC) may progress as 43% of patients with muscle invasive bladder cancer treated with radical cystectomy were initially diagnosed with NMIBC (3). Therefore, identifying NMIBC patients in whom the cancer is more likely to progress is an important issue to consider for in the management of these patients. A risk Table published by the European Organization for Research and Treatment of Cancer (EORTC) previously suggested stratifying patients into low-, intermediate-, and high-risk groups, a strategy designed to help guide the management of these patients (4).

Recent studies have demonstrated the role of systemic inflammation in the development and progression of cancer (5). For example, an elevated neutrophil-to-lymphocyte ratio (NLR) has consistently been found to be associated with muscle-invasive disease, extravesical disease, along with worse overall and disease-free survival rates (6-9). Recently published data have also revealed that an elevated NLR is an independent predictor of disease progression and recurrence in patients with NMIBC (10). The aim of the current study was to evaluate whether preoperatively measurement of NLR can predict lamina propria invasion in patients with NMIBC.

## MATERIAL AND METHODS

This study was approved by the local ethics committee. We retrospectively reviewed the medical records of 304 consecutive and newly diagnosed patients with non muscle invasive bladder cancer treated with transurethral resection between January 2008 and June 2014. Patients with preoperative infection, hematological malignancies and unexplained leukocytosis (n=33) were excluded from the study. Patients were divided into two groups according to their pathological stage (Group 1: Ta, n=161; Group 2: T1, n=110). From each patient, we obtained preoperative blood data including white blood cell (WBC), neutrophil (N) and lymphocyte counts (L). NLR was calculated by dividing N by L. The size and number of tumors were determined using preoperative radiological imaging and cyctoscopic examination prior to transuretral resection. Groups were then compared with regard to age, gender, tumor size, tumor number, WBC, N, L, and NLR.

### Statistical analyses

Statistical analyses were performed using the Number Cruncher Statistical System (NCSS) (2007, Utah, USA). Categorical variables were summarized using actual counts and percentages, whereas continuous variables were summarized using the mean±SD. Parametric and non parametric variables were evaluated using the t-test and chi-squared test, respectively. Logistic regression analysis was also used to determine predictors of T1 stage tumors. Statistical significance was considered at p<0.05.

## RESULTS

In total, 271 patients were enrolled in this study (27 women and 244 men). Mean age was higher in Group 2 than in Group 1 (67.3±10.8 vs. 62.9±10.8, p<0.001). The incidence of high grade tumors and tumors ≥3cm in size was statistically higher in Group 2 than in Group 1 (70.9% vs. 9.9%, p=0.0001; 71.8% vs. 36%, p=0.0001, respectively). While the mean WBC and N counts were statistically insignificant (7.63±1.87 vs. 7.69±1.93, p=0.780; 4.72±1.54 vs. 4.46±1.38, p=0.140; respectively), L was significantly lower and NLR was significantly higher in Group 2 than in Group 1 (2.07±0.75 vs. 2.4±0.87, p=0.001; 2.62±1.5 vs. 2.19±1.62, p=0.029; respectively) (Table-1).

**Table 1:** Patients characteristics.

		Group 1 (Ta, n:161)		Group 2 (T1, n:110)		p
Age		62.9±10.8		67.3±10.77		0.001^¥^
Gender	Man (%)	146	90.7%	98	89.1%	0.667^★^
	Woman (%)	15	9.3%	12	10.9%	
**Grade**	PUNLMP (%)	51	31.7%	2	1.8%	0.0001^★^
	Low Grade (%)	94	58.4%	30	27.2%	
	High Grade (%)	16	9.9%	78	70.9%	
Tumor Size	<3 cm (%)	103	64%	31	28.2%	0.0001^★^
	≥3 cm (%)	58	36%	79	71.8%	
Tumor Number	Single Tumor (%)	107	66.5%	68	61.8%	0.443^★^
	Multiple Tumor (%)	54	33.5%	42	38.2%	
WBC (mean±sd)		7.69±1.93		7.63±1.87		0.780^¥^
Neutrophil (mean±sd)		4.46±1.38		4.72±1.54		0.140^¥^
Lymphocyte (mean±sd)		2.4±0.87		2.07±0.75		0.001^¥^
NLR (mean±sd)		2.19±1.62		2.62±1.5		0.029^¥^

**PUNLMP =** Papillary urothelial neoplasm of low malignant potential **WWBC =** White blood cell; **NLR =** Neutrophil-to-lymphocyte ratio. ¥ = Independent sample t test; ★ = chi-square test

In order to evaluate the factors that might affect the presence of T1 tumors, we performed logistic regression analysis. High grade tumor (p=0.001), tumor size (≥3cm) (p=0.001), and L (p=0.049) were all associated with T1 tumors (Table-2).

**Table 2:** Logistic regression analyses for predicting T1 tumors.

	B	S.E.	p	OR	OR %95 CI	

					Lower	Upper
Grade			**0.001**			
Grade (Low Grade)	-4.63	0.78	**0.001**	0.01	0.00	0.05
Grade (High Grade)	2.62	0.36	**0.001**	0.07	0.04	0.15
Tumor size (≥3cm)	1.21	0.35	**0.001**	0.30	0.15	0.59
Lymphocyte	-0.41	0.21	**0.049**	0.66	0.44	1.00

**Figure 1:**
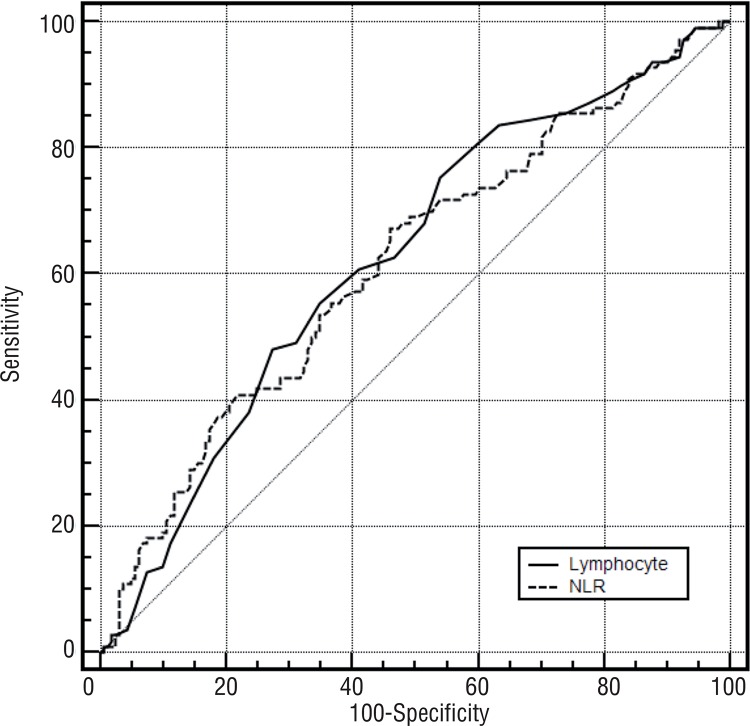
Assesment of cut off value of neutrophil-to-lymphocyte ratio and lymphocyte counts to predict T1 tumors.

According to the receiver operating characteristic analysis, the optimum cut-off value for NLR and L was >1.84 (area under curve [AUC] 0.616, 95% CI, 0.556-0.675) and ≤2.4 ([AUC] 0.625, 95% CI, 0.564-0.683), respectively (Figure-1). Sensitivity for NLR and L was 67.3% vs. 75.5%, whereas specificity was 54.1% vs. 47%, respectively (Table-3).

**Table 3:** Sensitivity and specificity for NLR and lymphocyte.

Cut off	Sensitivity	Specificity	PPV	NPV	+LR
> 1.84 NLR	67.27	54.04	50.0	70.7	1.46
≤ 2.4 Lymphocyte	75.45	46.96	48.8	73.3	1.40

**NLR =** Neutrophil-to-lymphocyte ratio; **PPV =** Positive predictive value; **NPV =** Negative predictive value.

## DISCUSSION

Non-muscle invasive bladder cancer represents a heterogeneous group of tumors with differential rates of recurrence and progression (11). The risk of progression primarily depends on the tumor grade, number, and size of tumor (12). Recently published data showed that the EORTC risk model is useful in predicting the progression of NMIBC but also noted the importance of updating new risk markers to improve the risk classification and prediction of progression (13). The current study assessed the predictive value of preoperative blood tests for lamina propria invasion in patients with NMIBC who were treated with TUR. Our analyses showed that low L (≤2.4) can act as an effective predictor for T1 tumors. While NLR was unable to predict T1 tumors with statistical probability, NLR was statistically higher in patients with T1 tumors than in patients with Ta tumors. Furthermore patients with NLR >1.8 exhibited a risk of developing a T1 tumor that was 1.5 times greater.

Increasing evidence supports the involvement of systemic inflammation in the growth and progression of tumors (5). NLR, is a parameter of stress and systemic inflammation, which is readily measurable in substantially ill patients (14). The association of pretreatment NLR and prognosis has also been reported for various other types of cancer, including ovarian cancer, gastric cancer, hepatocellular carcinoma, non-small cell lung cancer, and bladder cancer (15-20). Moreover, preoperative NLR was found to be associated with the progression and recurrence of NMIBC (7, 10, 20).

The relationships between increased NLR, and poor prognosis, and advanced stage of tumor can be explained by the impaired immune response of hosts to the tumor, which is dependent on L (14). Tumor proliferation and the survival of malignant cells ultimately depend on inflammation in the tumor microenvironment, and inflammation is also known to stimulate tumor angiogenesis, invasion, and metastasis (5). In advanced cancer patients, a variety of biological factors such as leukocytosis, lymphocytopenia, and C-reactive protein have been identified as having a definite correlation with prognosis (21). Because neutrophils inhibit the immune system and lymphocytes have a role in cell-mediated immunity in the host, such cellular components may reflect the host inflammatory and immune response (22). This hypothesis was confirmed by the fact that we observed a lower L among patients with T1 tumors in the present study.

As a result of exposure to carcinogens, the accumulation of mutations, and a reduction in immune function, the incidence of cancer and the prevalence of more advanced tumor stages are more common in elderly patients (23). Aged patients are susceptible to a range of changes that serve to diminish immune function such as a reduction in the number of functional B and T cells, modifications in the production and secretion of cytokines, reduced cytotoxic activity of CD8^+^ T cells, qualitative deficiency of B lymphocytes with reduced response to exogenous antigens, decline in the activity of natural killer cells, and possibly a deficiency in antigen-presenting cells (23). On the other hand, age does not appear to influence L (24). These circumstances indicate that the differences observed between our two study groups in terms of L and NLR cannot be explained by only age.

To the best of our knowledge this is the first study that evaluated whether preoperative measurement of NLR can predict lamina propria invasion in patients with NMIBC and demonstrated that NLR is statistically higher in T1 tumors than Ta. Moreover, low L counts can predict lamina propria invasion in NMIBC. Our results are likely to be beneficial to surgeons when explaining the risk of progression to patients with NMIBC prior to surgery.

The limitations of our present study include its retrospective nature and the relatively small number of patients studied. These factors may reduce the reliability of our preoperative results. Furthermore, the two groups being compared were statistically different in terms of age. This difference could be considered as a form of selection bias, although we suggest that this difference simply reflects the very nature of bladder cancer as it is well known that advanced age is related to advanced stages of bladder cancer. Moreover, the tumor characteristics were not well described in the operation notes and we had many missing data when we evaluated TURB records. Therefore, we used preoperative radiological imaging and cystoscopic examination together instead TURB records alone.

## CONCLUSIONS

The present study has indicated that high NLR and low L are associated with T1 stage, whereas a low L is able to predict lamina propria invasion in patients with NMIBC. These findings suggest that pretreatment NLR and L may provide valuable information in the clinical management of patients with NMIBC. Prospective studies between comparable groups are now required to validate the precise role of NLR as a potential risk factor for NMIBC.

## ARTICLE INFO

Int Braz J Urol. 2017; 43: 67-72
